# ERK signaling is required for VEGF-A/VEGFR2-induced differentiation of porcine adipose-derived mesenchymal stem cells into endothelial cells

**DOI:** 10.1186/s13287-017-0568-4

**Published:** 2017-05-12

**Authors:** Sami G. Almalki, Devendra K. Agrawal

**Affiliations:** Department of Clinical and Translational Science, Creighton University School of Medicine, Omaha, NE 68178 USA

**Keywords:** Adipose-derived mesenchymal stem cells, Endothelial cell differentiation, Vascular endothelial growth factor receptor type 2, Mitogen-activated protein kinase, Extracellular signal-regulated kinase 1/2, c-Jun NH2-terminal kinase, Stress activated protein kinase-2, Angiotensin type-2 receptor

## Abstract

**Background:**

Cell-based therapy that can rejuvenate the endothelium with stimulated adipose-derived mesenchymal stem cells (AMSCs) is a promising therapeutic strategy for the re-endothelialization of denuded arteries at the stenting site. Previously, we have shown that silencing of MMP-2 and MMP-14 inhibits vascular endothelial growth factor receptor type 2 (VEGFR2) cleavage, and induces differentiation of AMSCs toward the endothelial cell (EC) lineage. In this study, we examined the underlying signaling pathways that regulate differentiation of AMSCs to ECs in vitro through VEGFR2.

**Methods:**

AMSCs were isolated from porcine abdominal adipose tissue. The isolated AMSCs were characterized by positive expression of CD29, CD44, and CD90 and negative expression of CD11b and CD45. The isolated MSCs were transfected with siRNA to silence MMP-2, MMP-14, and angiotensin receptor 2 (ATR2). Cells were suspended either in endothelial basal media (EBM) or endothelial growth media (EGM) with various treatments. Flow cytometry was performed to examine the expression of EC markers, and western blot analysis was performed to examine the expression and activity of various kinases. Scratch assay was performed to examine the cell migration. Data were analyzed by ANOVA using PRISM GraphPad.

**Results:**

After 10 days of stimulation for EC differentiation, the morphology of AMSCs changed to a morphology similar to that of ECs. Silencing MMP-2 and MMP-14 resulted in significant decrease in the number of migrated cells compared with the EGM-only group. ATR2 siRNA transfection did not affect the migration and differentiation of AMSCs to ECs. Stimulation of AMSCs for EC differentiation with or without MMP-2 or MMP-14 siRNA resulted in significant increase in p-ERK, and significant decrease in p-JNK. There was no significant change in p-p38 in all three groups compared with the EBM group. ERK inhibition resulted in significant decrease in the expression of EC markers in the EGM, EGM + MMP-2 siRNA, and EGM + MMP-14 siRNA groups. The VEGFR2 kinase inhibitor induced a dose-dependent inhibition of ERK.

**Conclusion:**

The ERK signaling pathway is critical for VEGF-A/VEGFR2-induced differentiation of AMSCs into ECs. These findings provide new insights into the role of the ERK signaling pathway in AMSC differentiation to ECs for potential clinical use in cardiovascular diseases.

## Background

Atherosclerosis is the precursor of most common cardiovascular diseases (CVD), and is the leading cause of mortality all over the world [1]. The endothelium plays an important role in the development and progression of atherosclerosis. Endothelial dysfunction or loss of the endothelial layer is known as an early change in the wall of blood vessels that leads to progression of the atherosclerotic plaque [2]. Endothelial dysfunction is also a crucial factor in the development and progression of restenosis, as a result of high proliferation activity of vascular smooth muscle cells (VSMC) from the tunica media to the intima. Angioplasty and stenting techniques are widely used around the world for improving blood flow to the heart in cardiovascular diseases [3]. The high proliferation activity of VSMCs could be a result of acute injury located around stent struts [3]. The development of cell-based therapy for the treatment of vascular injuries has been increasing in the last few years using mesenchymal stem cells (MSCs) [4–6]. Cell-based therapy that can rejuvenate the endothelium with stimulated adipose-derived MSCs (AMSCs) is a promising therapeutic strategy for re-endothelialization at the site of intravascular stenting to prevent restenosis [7].

Identifying the molecular factors and mechanisms that regulate AMSC differentiation is important in the promotion of a greater understanding of these highly useful cells. Previously, we have reported that silencing matrix metalloproteinase (MMP)-2 and MMP-14 induces the differentiation of porcine AMSCs to endothelial cells (ECs) [8]. Also, MMP-2 and MMP-14 cleave vascular endothelial growth factor receptor type 2 (VEGFR2) and inhibit AMSC differentiation toward the endothelial lineage [8]. However, the downstream signaling pathways that regulate the differentiation of AMSCs to ECs through VEGFR2 are still undefined.

Among the intracellular signaling pathways that can be mediated by VEGFR2, the mitogen-activated protein kinase (MAPK) signaling pathways appear to be the primary candidate [9]. Extracellular signal-regulated kinase (ERK1/2), c-Jun NH2-terminal kinase (JNK), and stress activated protein kinase-2 (p38) are the members of the classical MAPK cascades [10]. MAPK pathways are well known as crucial signaling pathways in the differentiation of MSCs to various lineages [11–17]. The binding of VEGF-A to VEGFR2 phosphorylates PLC-γ, and activates the MAPK/ERK and p38 MAPK signaling pathways [9, 18]. The differentiation of bone marrow-derived MSCs (BM-MSCs) to ECs was found to be mediated by VEGF, which induces the phosphorylation of p42 MAPK/ERK [19]. Inhibition of ERK phosphorylation blocks the expression of EC markers and BM-MSC differentiation to ECs [19]. The stimulation of stem cells from exfoliated deciduous teeth with EC differentiation media induces the activation of ERK, and inhibition of ERK blocks the differentiation to ECs [20]. The inhibition of p38, but not ERK, restrains the differentiation of BM-MSCs to osteoblasts [21]. The osteogenic differentiation of mesenchymal progenitor cells requires the activity of p38, and ERK inhibition activates BMP9-induced osteogenic differentiation [22]. JNK was also found to promote target gene expression during stem cell differentiation to neuronal cells [23]. Moreover, JNK inhibition was found to prevent the neuronal differentiation of mouse embryonic stem cells [24]. The activities of JNK and p38 are required for the differentiation of MSCs to osteocytes [25].

The effect of angiotensin II (Ang II) was implicated in the physiological and pathophysiological events related to the cardiovascular system [26]. Ang II has a key role in the migration of MSCs to the site of injury [26]. Ang II in combination with VEGF was found to induce BM-MSC differentiation to ECs, and the blockage of angiotensin type-2 receptor (ATR2) attenuated this process [27]. Stimulation of ATR2 also activates neural differentiation, and monocyte differentiation to dendritic cells [28, 29]. Therefore, the role of ATR2 in the differentiation of AMSCs to ECs was investigated.

In this study, we examined the signaling events downstream of VEGFR2. Our findings show that ERK, but not p38 or JNK, induces the differentiation of AMSCs to ECs. The results also indicate that ATR2 activity is not required for EC differentiation of AMSCs. These findings provide new insights into the role of the ERK signaling pathway in the differentiation of AMSCs into ECs.

## Methods

### Isolation and culture of porcine AMSCs

AMSCs were isolated from porcine adipose tissue as described previously [8, 30]. Briefly, porcine adipose tissue was collected from the abdominal wall of pigs from a local slaughterhouse (Hormel, Fremont, NE, USA), and transferred to the laboratory in sterile conditions in Dulbecco’s modified Eagle medium (DMEM; Invitrogen, Grand Island, NY, USA) with the antibiotics 100 mg/ml penicillin (Sigma–Aldrich, St. Louis, MO, USA), 100 mg/ml streptomycin (Sigma–Aldrich), and 2 mM Glutamax (Invitrogen, Carlsbad, CA, USA). The transferred abdominal adipose tissue was washed with phosphate-buffered saline (PBS) and minced into small pieces with sterile scissors. The small minced pieces were then digested with 15 ml of 0.2% type-1 collagenase (Sigma–Aldrich) for 2 hours at 37 °C. DMEM containing 10% fetal bovine serum (FBS) (Gibco, USA) was added to stop the collagenase activity, followed by centrifugation at 400 × *g* for 10 minutes to separate the floating cells from the vascular stromal fraction. The pellets were then resuspended in serum-complete medium (DMEM, 10% FBS, 5% penicillin/streptomycin, and 1% Glutamax), and filtered through a 100-μm nylon mesh strainer to remove any undigested tissue. The filtered cells were carefully added to 50-ml tubes in the top of a 1.077 g/ml histopaque (Sigma-Aldrich) for density gradient centrifugation at 400 × *g* for 30 minutes. The enriched cells were then collected from the interphase and washed twice with serum-free medium. The pellets were finally resuspended in DMEM containing 10% FBS, 100 mg/ml penicillin/streptomycin, and 2 mM Glutamax, and were cultured in a 25-cm^2^ flask at 37 °C with 5% CO_2_/95% air and 90% relative humidity. Nonadherent hematopoietic cells were removed by medium change every 24 hours for 3 days. Thereafter, the medium was changed every 3 days. Once adherent AMSCs became confluent, they were trypsinized using 0.25% Trypsin–EDTA (Sigma-Aldrich) and transferred to fresh 25-cm^2^ culture flasks. All experiments were performed using MSCs at three to six passages.

### Characterization of AMSCs

#### Immunophenotyping

AMSCs at three to six passages were trypsinized and flow cytometric analysis was performed to examine the expression of AMSC markers CD29, CD44 and CD90, and negativity for the hematopoietic stem cell marker CD45 and macrophage marker CD11b. Cells were detached from the monolayer with 0.25% Trypsin–EDTA, and washed twice with PBS containing 4% FBS. The AMSCs were then incubated for 1 hour at 4 °C in the dark with conjugated monoclonal antibodies against CD11b, CD45, CD29, CD44, and CD90 (eBiosciences, CA, USA). The dilutions of the antibodies were according to the specifications of the manufacturers. The cells were washed three times in PBS, and resuspended in 500 μl PBS. Flow cytometry was performed on a FACS Aria Flow Cytometry System (BD Biosciences, San Jose, CA, USA). Fluorochrome-labeled IgG (eBiosciences) served as the isotype control as well as positive and negative beads (OneComp eBeads; eBiosciences).

AMSCs were stimulated for EC differentiation for 10 days. The cells were then detached from the monolayer with 0.25% Trypsin–EDTA, and harvested for flow cytometry analysis to identify the EC markers CD31 and CD144. Direct conjugated monoclonal antibodies were used against CD31 and CD144 (17-0319, 25-1449; eBiosciences).

#### Differentiation of AMSCs to ECs

The differentiation process started at 50–60% confluency of AMSCs. The AMSC culture was stimulated with endothelial growth medium (EGM) composed of Endothelial Basal Medium-2 (Gibco, Grand Island, NY, USA), growth supplements (containing hydrocortisone, human fibroblast growth factor (hFGF-b), R3-insulin-like growth factor-1 (R3-IGF-1), ascorbic acid, human epithelial growth factor (hEGF), GA-1000, heparin), 2% FBS (EGM-2 Bullet Kit; Lonza, Walkersville, MD, USA), and 50 ng/ml VEGF-165 (Peprotech, Rocky Hill, NJ, USA). The cells were maintained at 37 °C with 5% CO_2_/95% air and 90% relative humidity, and the medium was changed every 3 days. The cells were detached from the monolayer with 0.25% Trypsin–EDTA, and collected for analysis after 10 days of stimulation for EC differentiation.

Human umbilical vein endothelial cells (HUVECs) (ATCC® PCS-100-010™; ATCC) were used as a positive control.

#### Migration assay

The in-vitro scratch assay was performed to examine cell migration. Cells were plated into a 12-well cell culture plate, and allowed to grow in serum-complete medium to confluence with or without MMP-2 siRNA, MMP-14 siRNA, or ATR2 siRNA. Cells were then washed, and starved for 24 hours in serum-free medium to minimize cell proliferation. A sterile pipette tip was used to make a 1-mm wide scratch across the cell layer. Cells were then washed extensively with PBS to remove cellular debris and floating cells before adding media with different treatments. Plates were photographed immediately after scratching, and after 24 hours at the same location of initial image under a bright-field microscope.

#### Cell transfection

The siRNA transfection of differentiating AMSCs was performed according to the manufacturer’s protocol. Briefly, AMSCs were plated in DMEM complete medium into T-25 flasks, and allowed to reach 50–60% confluency. The cells were then incubated with smartpool on-target plus siRNAs for MMP-2, MMP-14, or ATR2 (4313, 4323, and 24182; Dharmacon, USA). A final siRNA concentration of 50 nM was mixed with 10 μl DharmaFECTTM-1 (Dharmacon), and allowed to complex by incubation for 10 minutes at room temperature. The transfection mixture was then applied to the AMSCs in a total volume of 4 ml of EGM, and incubated at 37 °C in 5% CO_2_. Cell viability and the capacity for differentiation were not affected under these conditions.

#### Western blot analysis

After 10 days of differentiation with or without siRNA transfection, the total protein lysates were isolated and quantified. Briefly, the cell pellet was resuspended in 100 μl of RIPA buffer (Sigma, USA) and 1 μl of protease and phosphatase inhibitor cocktail (78443; ThermoScientific, USA). The cell lysates were then vortexed, and incubated in ice for 10 minutes. This step was repeated three times. The supernatants were collected by centrifugation at 14,000 × *g* for 15 minutes. Total protein lysates were quantified by Enspire Manager Software (PerkinElmer Enspire, USA).

The protein was separated by SDS-PAGE and transferred onto a nitrocellulose membrane (Bio-Rad, USA). The membrane was incubated for 1 hour in blocking solution (1× TBS, pH 7.6, 0.1% Tween-20, and 7% BSA). The membrane was then incubated with a primary antibody to detect ATR2 (ab19134; Abcam, USA), phospho-ERK (4370; Cell Signaling, USA), ERK (sc-94; Santa Cruz, USA), phospho-JNK (4668; Cell Signaling), JNK (sc-571; Santa Cruz), phospho-p38 (4511; Cell Signaling), p38 (9212; Cell Signaling), and GAPDH (NB300-221; Novus, USA) overnight. After washing, the membrane was then incubated with HRP-conjugated secondary antibody (1:1000) in blocking solution for 1 hour at room temperature. HRP activity was detected by incubating the membrane in a chemiluminescence kit (Pierce, USA). Image Lab Software (Bio-Rad) was used for imaging and densitometric analysis.

#### ERK inhibition

The phosphorylation of ERK1/2 was inhibited by ERK inhibitor U0126 (662005; Calbiochem, USA) to analyze the role of the ERK pathway in the differentiation of AMSCs to ECs before and after MMP-2 or MMP-14 silencing. AMSCs were plated in DMEM complete medium in T25 flasks, and allowed to reach 50–60% confluency. ERK inhibitor was applied to the AMSCs in EGM at a final concentration of 5 μM. To examine the phosphorylation status of ERK, differentiated AMSCs were incubated in EGM and ERK inhibitor for 3 hours, and tested for ERK activity by western blot analysis. Cell viability was not affected under these conditions.

#### VEGFR2 inhibition

VEGFR2 was inhibited by VEGFR2 Tyrosine Kinase Inhibitor (676499; Calbiochem) to analyze its role in the differentiation of AMSCs to ECs before and after MMP-2 and MMP-14 silencing. AMSCs were plated in DMEM complete medium in T25 flasks, and allowed to reach 50–60% confluence. VEGFR2 kinase inhibitor was applied to the AMSCs in EGM at a final concentration of 5 μM. Cell viability was not affected under these conditions.

### Statistical analysis

Statistical analysis was performed using GraphPad Prism. Multiple group comparisons were performed by Bonferroni’s multiple comparison test using one-way ANOVA. Descriptive data are presented as the mean ± standard deviation (SD). Differences were considered significant at *p* < 0.05.

## Results

### AMSC characterization

**Fig. 1:**
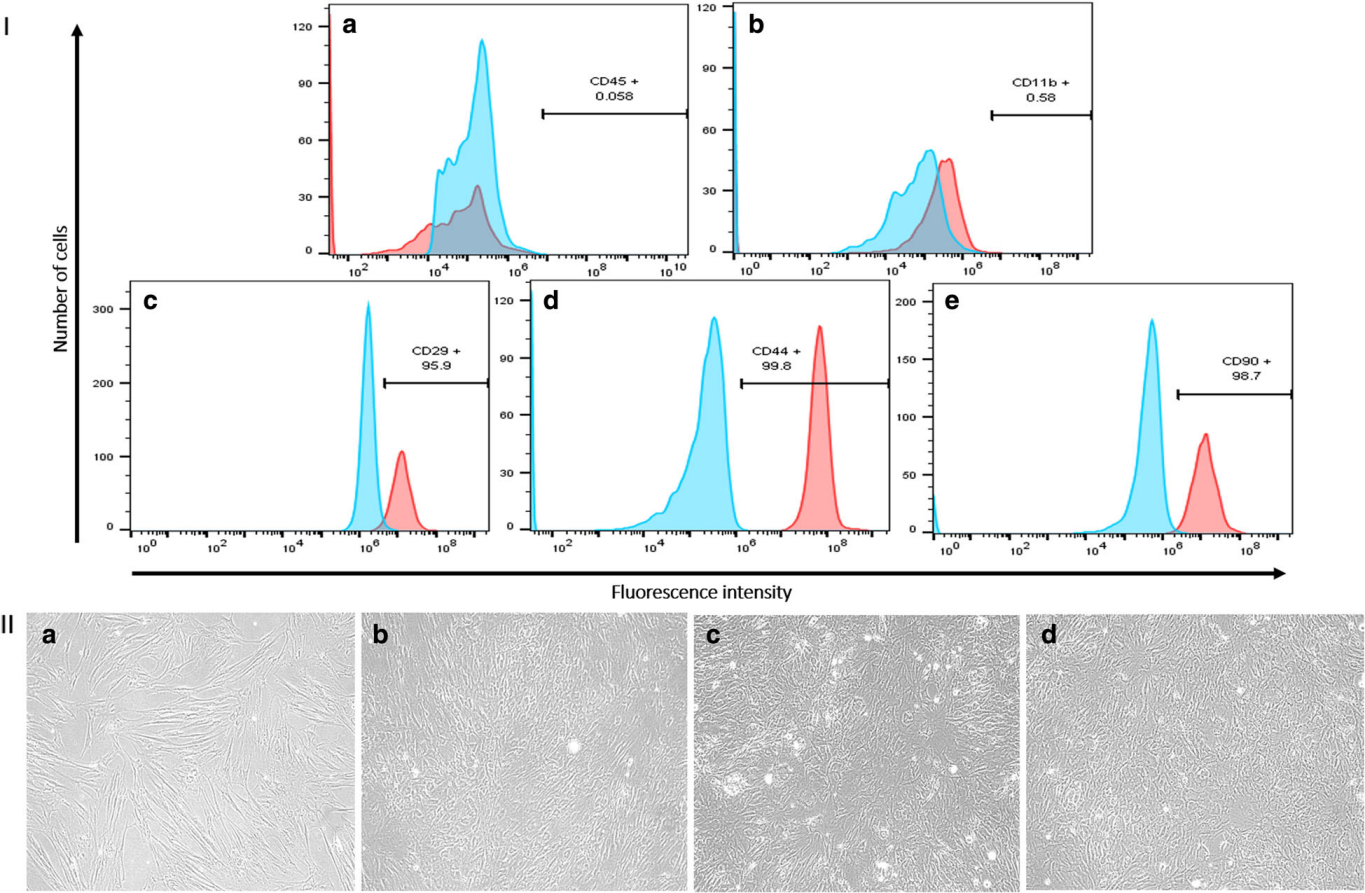
Immunophenotyping of AMSCs and differentiation to ECs. **I** Immunophenotyping of AMSCs. Flow cytometry data showed no expression for CD45 (*A*) and CD11b (*B*), and high expression for the MSC markers CD29 (*C*), CD44 (*D*), and CD90 (*E*). *Blue peaks*, profile of the isotype control. Flow cytometry was carried out on a FACS Aria Flow Cytometry System. **II** Endothelial cell differentiation. (*A*) AMSCs in culture with DMEM. Endothelial cell differentiation after 10 days of stimulation with EGM (*B*), EGM + MMP-2 siRNA (*C*), and EGM + MMP-14 siRNA (*D*). Change in the morphology of cells after 10 days of stimulation for endothelial cell differentiation (Color figure online)

Primary cultures of AMSCs were isolated from porcine abdominal fat. The adherent cells showed fibroblast-like morphology typical of MSCs (Fig. 1II-A). Flow cytometry analysis was performed to examine the expression of MSC markers in the isolated porcine AMSCs. The cells expressed CD29 (Fig. 1I-C), CD44 (Fig. 1I-D), and CD90 (Fig. 1I-E), indicating that the cells originate from a mesenchymal lineage. Contamination with other cells was ruled out by negative immunoreactivity to CD11b (macrophage marker) (Fig. 1I-B) and CD45 (hematopoietic stem cell marker) (Fig. 1I-A). The daily change of medium after seeding the isolated cells was effective in eliminating the CD11b^+^ and CD45^+^ cell populations in AMSC culture.

### EC differentiation

The isolated AMSCs were stimulated for EC differentiation in EGM medium containing 50 ng/ml (1.75 nM) of VEGF with or without MMP-2 siRNA, MMP-14 siRNA, or ATR2 siRNA. After 10 days of stimulation, the morphology of AMSCs in EGM (Fig. 1II-B), EGM + MMP-2 siRNA (Fig. 1II-C), and EGM + MMP-14 siRNA (Fig. 1II-D) changed to a morphology similar to that of ECs.

### Migration assay

**Fig. 2:**
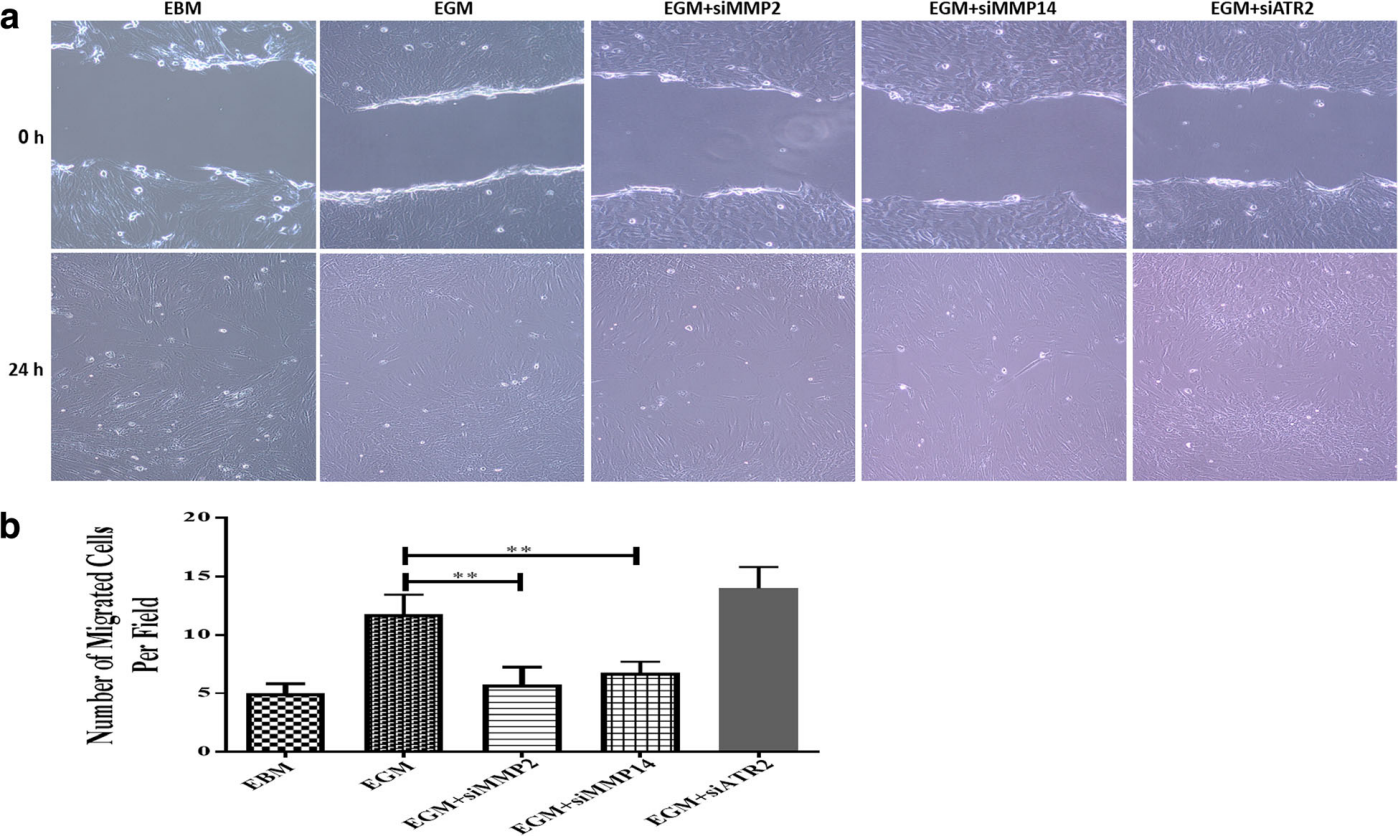
Migration assay. AMSCs in EBM, EGM, EGM + MMP-2 siRNA, EGM + MMP-14 siRNA, and EGM + ATR2 siRNA showed migration activity after 24 hours of making the scratches (**a**). MMP-2 and MMP-14 siRNA resulted in significant decrease in the number of migrated cells per field compared with EGM-only cells, whereas ATR2 siRNA silencing had no effect on the migration activity of AMSCs during EC differentiation (**b**). **p < 0.01. *EBM* endothelial cell basal medium, *EGM* endothelial cell growth medium, *MMP* matrix metalloproteinase, *ATR2* angiotensin receptor R2

AMSCs were examined to evaluate the effect of MMP-2, MMP-14, and ATR2 siRNA silencing on their migratory activity during EC differentiation (Fig. 2a). After 24 hours, silencing MMP-2 and MMP-14 resulted in significant decrease in the number of migrated cells compared with EGM-only cells (Fig. 2b). However, ATR2 siRNA silencing did not affect the migration activity of AMSCs during EC differentiation (Fig. 2b). AMSCs also showed migration activity, but less than that of differentiated AMSCs (Fig. 2b).

### Cell transfection

**Fig. 3:**
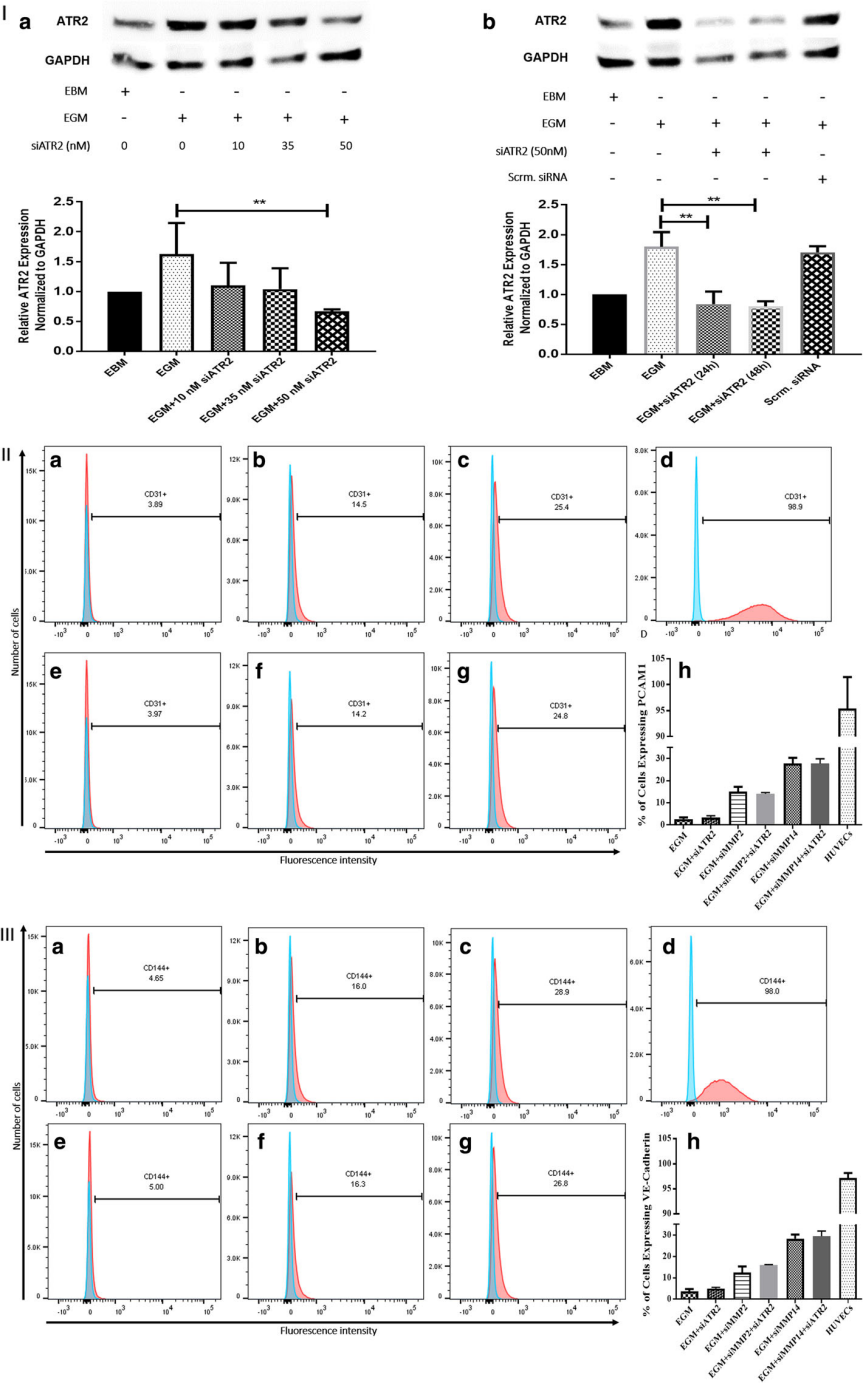
ATR2 siRNA transfection and immunophenotyping for EC markers. **I** Concentration selection for siRNA transfection. Three different concentrations (10, 35, and 50 nM) of ATR2 siRNA were used according to the manufacturer’s protocol. Western blot analysis showed inhibition of ATR2 by 10, 35, and 50 nM of ATR2 siRNA. However, 50 nM of ATR2 siRNA showed the highest inhibition among all three different concentrations (*A*). ATR2 silencing by siRNA transfection with EGM compared with AMSCs with EGM and EGM + scrambled siRNA (negative control) (*B*). GAPDH was used as a housekeeping gene. **II** Flow cytometric analysis of PECAM1 (CD31) in four different groups; control group with EGM (*A*), AMSCs with EGM and MMP-2 siRNA (*B*), AMSCs with EGM and MMP-14 siRNA (*C*), and HUVECs as the positive control (*D*). Cell transfection with 5 μM of ATR2 siRNA for EGM (*E*), AMSCs with EGM and MMP-2 siRNA (*F*), and AMSCs with EGM and MMP-14 siRNA (*G*). Flow cytometry data were analyzed to show the significant differences between the groups (*H*). **III** Flow cytometric analysis of VE-cadherin (CD144) in four different groups: control group AMSCs with EGM (*A*), AMSCs with EGM and MMP-2 siRNA (*B*), AMSCs with EGM and MMP-14 siRNA (*C*), and HUVECs as the positive control (*D*). Cell transfection with 5 μM of ATR2 siRNA for EGM (*E*), AMSCs with EGM and MMP-2 siRNA (*F*), and AMSCs with EGM and MMP-14 siRNA (*G*). Flow cytometry data were analyzed to show the significant differences between the groups (*H*). **p < 0.01. *EBM* endothelial cell basal medium, *EGM* endothelial cell growth medium, *MMP* matrix metalloproteinase, *ATR2* angiotensin receptor R2, *HUVEC* human umbilical vein endothelial cell

MMP-2 and MMP-14 siRNA transfections were carried out as described previously [8]. Similarly, to determine the concentration of siRNA for ATR2 silencing, we used three different concentrations of 10, 35, and 50 nM according to the manufacturer’s protocol. All three concentrations of ATR2 siRNA reduced the protein expression of ATR2. However, statistically significant inhibition was found only with 50 nM of ATR2 siRNA (Fig. 3I-A). ATR2 was then silenced with 50 nM of siRNA for further examinations of EC differentiation after ATR2 silencing. Western blot data showed that the expression of ATR2, after siRNA transfection for 24 and 48 hours, resulted in more than 50% inhibition compared with AMSCs in EGM and AMSCs in EGM + scrambled siRNA (Fig. 3I-B). An automated hemocytometer (Countess; Invitrogen, USA) with trypan blue staining was used to examine the cell viability after siRNA transfection. The cell viability was not affected by ATR2 siRNA transfection.

### Expression of EC markers after ATR2 siRNA

Flow cytometric analysis was performed to examine the expression of EC markers after 10 days of EC differentiation. AMSCs were cultured in EGM (Fig. 3II-A, III-A), EGM + MMP-2 siRNA (Fig. 3II-B, III-B), and EGM + MMP-14 siRNA (Fig. 3II-C, III-C), and with ATR2 siRNA for all three groups (Fig. 3II-E–II-G, III-E–III-G). Silencing ATR2 in the four groups showed no significant difference in the expression of EC markers PECAM1 (Fig. 3II-H) and VE-cadherin (Fig. 3III-H), compared with the same groups without ATR2 siRNA. AMSCs stimulated with EGM with or without ATR2 siRNA showed significant increases in the expression of both markers to about 5% in both groups (Fig. 3II-H, III-H). ATR2 silencing in the MMP-2 siRNA + EGM and MMP-14 siRNA + EGM groups showed significant increases in the expression of PECAM1 and VE-cadherin like that of the same groups without ATR2 siRNA (by about 13 and 28%, respectively, compared with the EGM only group) (Fig. 3II-H, III-H). HUVECs were used as a positive control (Fig. 3II-D, III-D).

### ERK, JNK, and p38 after 10 days of differentiation

To examine the role of ERK, JNK, and p38 in the differentiation of AMSCs to ECs, cell lysates were analyzed by western blot analysis to detect p-ERK, p-JNK, and p-p38 in AMSCs cultured with EBM, EGM, EGM + MMP-2 siRNA, and EGM + MMP-14 siRNA.

**Fig. 4:**
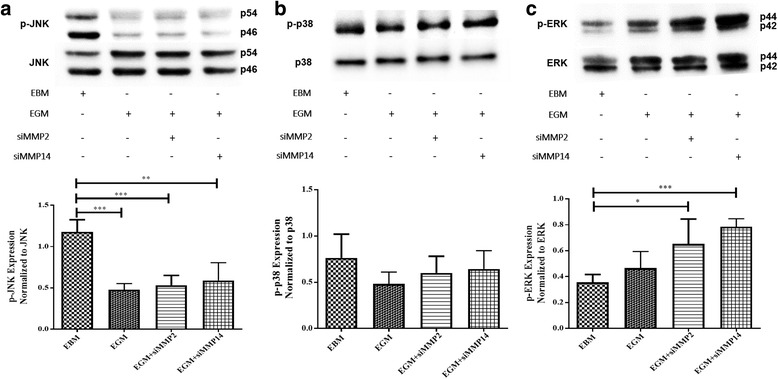
Phosphorylation of JNK, p38, and ERK after 10 days of AMSC differentiation to ECs. Western blot detection of p-JNK (**a**), p-p38 (**b**), and p-ERK1/2 (**c**) in AMSC lysates after 10 days of AMSC stimulation for EC differentiation with EBM (control), EGM, EGM + MMP-2 siRNA, and EGM + MMP-14 siRNA. Phospho-proteins were normalized to their total protein expressions. *p < 0.05, **p < 0.01, ***p < 0.001. *EBM* endothelial cell basal medium, *EGM* endothelial cell growth medium, *MMP* matrix metalloproteinase, *ERK* extracellular signal-regulated kinase, *JNK* c-Jun NH2-terminal kinase, *p38* stress activated protein kinase-2

After 10 days, the results showed no significant change in the phosphorylation of p38 in the EGM, EGM + MMP-2 siRNA, and EGM + MMP-14 siRNA groups compared with the EBM group (Fig. 4a). AMSCs cultured with EGM, EGM + MMP-2 siRNA, and EGM + MMP-14 siRNA showed significant decrease in the phosphorylation of JNK to less than 60% compared with the EBM group (Fig. 4b). AMSCs in EGM showed little increase in p-ERK in comparison with the EBM group (Fig. 4c). AMSCs cultured with EGM + MMP-2 siRNA showed significantly higher phosphorylation for ERK compared with the EBM group (Fig. 4c). Likewise, AMSCs cultured with EGM + MMP-14 siRNA showed greater significant increase in p-ERK compared with that of the EBM group (Fig. 4c).

### ERK inhibition

**Fig. 5:**
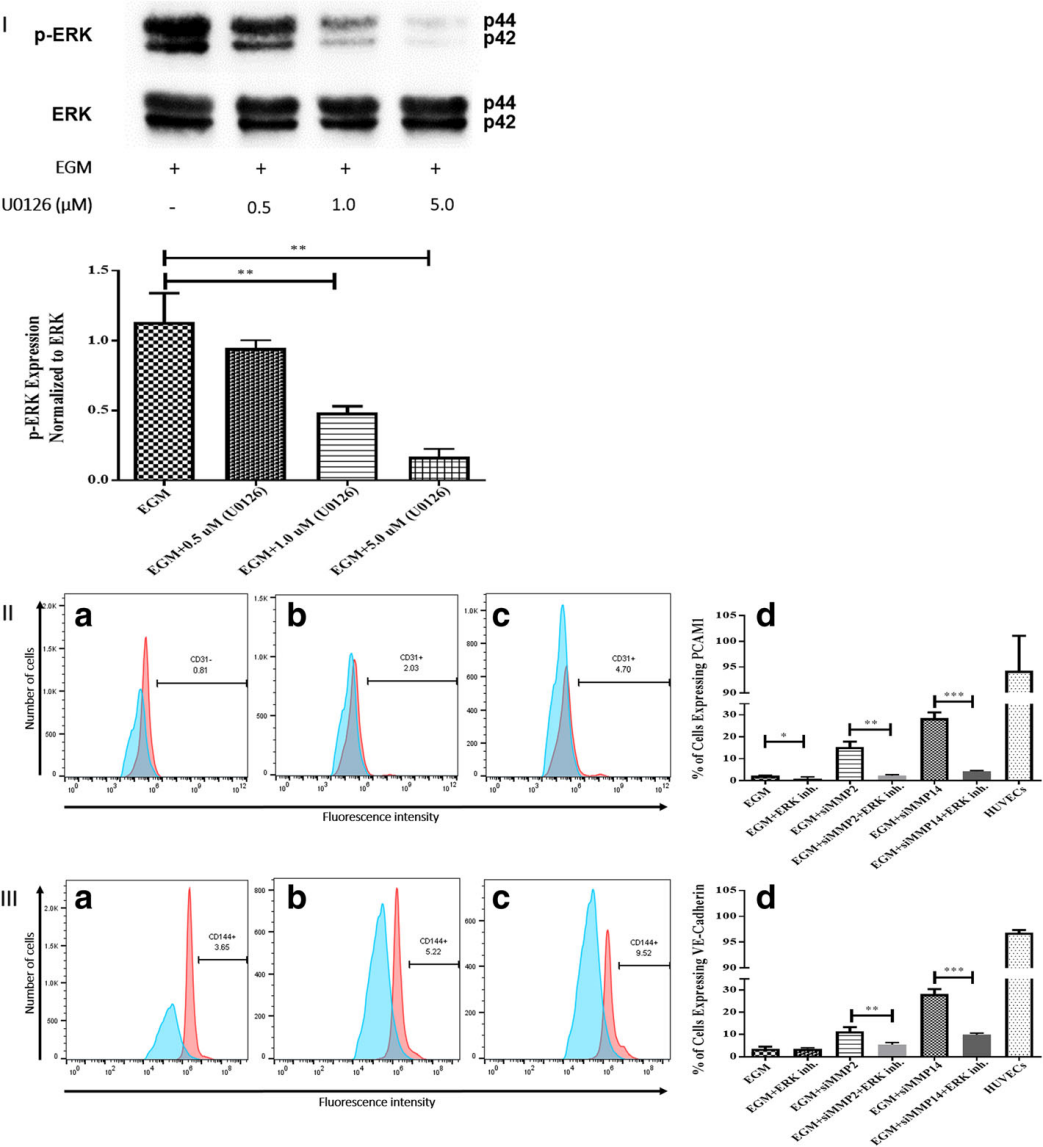
Inhibition of ERK phosphorylation and immunophenotyping for EC markers. **I** Concentration -dependent effect of ERK inhibitor (U0126). Three different concentrations (0.5, 1.0, and 5.0 μM) of U0126 were used. Western blot analysis showed significant inhibition of p-ERK by 1.0 and 5.0 μM of U0126. However, 5.0 μM of U0126 showed the highest inhibition among all three different concentrations. Phospho-ERK was normalized to its total protein expression. **II** Flow cytometric analysis of PECAM1 (CD31) with ERK inhibitor (U0126). Three different groups treated with 5.0 μM of U0126: AMSCs with EGM (*A*), AMSCs with EGM and MMP-2 siRNA (*B*), and AMSCs with EGM and MMP-14 siRNA (*C*). Flow cytometry data were analyzed to show the significant differences between the groups (*D*). **III** Flow cytometric analysis of VE-cadherin (CD144) with ERK inhibitor (U0126). Three different groups were treated with 5.0 μM of U0126: AMSCs with EGM (*A*), AMSCs with EGM and MMP-2 siRNA (*B*), and AMSCs with EGM and MMP-14 siRNA (*C*). Flow cytometry data were analyzed to show the significant differences with or without U0126 (*D*). *p < 0.05, **p < 0.01, ***p < 0.001. *EBM* endothelial cell basal medium, *EGM* endothelial cell growth medium, *MMP* matrix metalloproteinase, *ERK* extracellular signal-regulated kinase, *HUVEC* human umbilical vein endothelial cell

ERK was inhibited during AMSC differentiation to ECs by U0126 (662005; Calbiochem) to analyze its role in the differentiation process. For concentration selection of ERK inhibitor, three different concentrations of U0126 were chosen to determine the most effective concentration (0.5, 1.0, and 5.0 μM). The results showed significant decrease in the phosphorylation of ERK in EGM with 1.0 and 5.0 μM of U0126 compared with the EGM group (Fig. 5I). However, 5.0 μM of U0126 showed the highest inhibition among all three different concentrations (Fig. 5I). Cell viability was tested using an automated hemocytometer (Countess; Invitrogen) with trypan blue staining, and the results showed a high cell viability rate (94–97%) after ERK inhibition.

### Expression of EC markers after ERK inhibition

Flow cytometric analysis was performed to determine the role of ERK in the differentiation of AMSCs to ECs. AMSCs cultured with EGM, EGM + MMP-2 siRNA, and EGM + MMP-14 siRNA, with 5.0 μM of ERK inhibitor (U0126), were examined for the expression of EC specific markers PECAM1 and VE-cadherin. The EGM group treated with ERK inhibitor showed significant decrease for PECAM1 (Fig. 5II-A) compared with EGM only (Fig. 5II-D). There was no significant change in the expression of VE-cadherin after ERK inhibition (Fig. 5III-A) compared with the EGM group (Fig. 5III-D). Moreover, siRNA silencing of MMP-2 + EGM and ERK inhibitor resulted in significant decrease in the expression of both markers (Fig. 5II-B, III-B) in comparison with the same group without ERK inhibitor (Fig. 5II-D, III-D). Similarly, EGM + MMP-14 siRNA treated with ERK inhibitor showed significant decrease in the expression of PECAM1 and VE-cadherin (Fig. 5II-C, III-C) compared with the untreated group (Fig. 5II-D, III-D).

### Dose-dependent inhibition of ERK by VEGFR2 kinase inhibitor

**Fig. 6:**
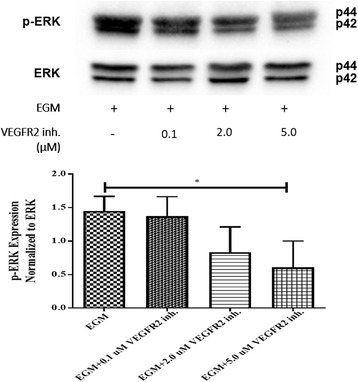
Dose-dependent inhibition of ERK by VEGFR2 kinase inhibitor. Western blot analysis of p-ERK with three different concentration of VEGFR2 kinase inhibitor (0.1, 2.0, and 5.0 μM), showed significant decrease in ERK phosphorylation with 5.0 μM of VEGFR2 kinase inhibitor. Phospho-ERK was normalized to its total protein expression. *p < 0.05. *EBM* endothelial cell basal medium, *EGM* endothelial cell growth medium, *ERK* extracellular signal-regulated kinase, *VEGFR2* vascular endothelial growth factor receptor-2

Our previous study demonstrated that VEGFR2 activity is required for AMSC differentiation to ECs, and inhibition of VEGFR2 resulted in significant decrease in the expression of EC markers (PECAM1 and VE-cadherin) [8]. Western blot analysis was performed to further confirm that ERK activity is dependent on upstream signals received from VEGFR2. The phosphorylation of ERK was examined after treatment with 0.1, 2.0, and 5.0 μM of VEGFR2 kinase inhibitor. AMSCs treated with 0.1 and 2.0 μM of VEGFR2 kinase inhibitor resulted in no significant change at the level of ERK phosphorylation compared with AMSCs in EGM only (Fig. 6). Treatment of the cells with 5.0 μM VEGFR2 kinase inhibitor showed significant decrease at the level of p-ERK compared with the EGM-only group (Fig. 6).

## Discussion

**Fig. 7:**
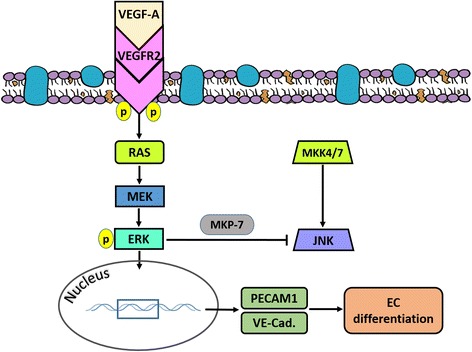
Signaling transduction pathway of AMSC differentiation to ECs and negative crosstalk between ERK and JNK. ERK receives signals from VEGFR2, and induces the transcription of EC markers during AMSC stimulation for EC differentiation. ERK activation phosphorylates MKP-7 and blocks the JNK signaling pathway. *ERK* extracellular signal-regulated kinase, *VEGF* vascular endothelial growth factor, *VEGFR2* vascular endothelial growth factor receptor-2, *JNK* c-Jun NH2-terminal kinase, *MKP-7* MAP kinase phosphatase-7, *EC* endothelial cell

There are numerous challenges to fully understand the mechanisms underlying the differentiation of AMSCs to ECs, including signaling pathways and cross-talk between them that induce AMSC differentiation toward a specific lineage. In this study, CD11b^–^/CD45^–^ AMSCs were immunopositive for CD44, CD90, and CD29, which are known to be expressed in MSCs isolated from adipose tissue [31–33]. The high percentage of isolated cells that expressed CD29, CD44, and CD90 (96, 99, and 97%, respectively) indicated that the cultured cells originate from a mesenchymal lineage, and suggested the high purity of the AMSCs in culture. Contamination with other cells was ruled out by negative immunoreactivity to CD11b (macrophage marker) and CD45 (hematopoietic stem cell marker). The daily change of medium after seeding the isolated cells was effective in eliminating the CD11b^+^ and CD45^+^ cell populations and other nonadherent cells in AMSC culture. We demonstrate that ERK activity is required for the upstream signals from VEGFR2 to induce the EC differentiation of AMSCs (Fig. 7). The inhibition of ERK resulted in significant decrease in the expression of CE markers, and VEGFR2 kinase inhibitor decreases the phosphorylation of ERK. However, silencing ATR2 did not affect the expression of EC markers after 10 days of stimulation for EC differentiation.

Cell migration requires molecular and structural changes in order for the cell to move from an adhesive to a migratory state [34]. We have recently shown that silencing MMP-2 and MMP-14 induces the expression of EC markers and endothelial functionality [8]. Herein, we examined the effect of MMP-2 and MMP-14 inhibition on the migration of AMSCs during EC differentiation. MMPs have been identified by their ability to translocate from the membrane or cytosol to the nucleus, playing important roles in cell development, morphogenesis, migration, proliferation, and differentiation [35, 36]. A broad-spectrum MMP inhibitor inhibited the migration of MSCs, indicating critical roles for MMPs in this process [13]. MMP-2 knockdown resulted in the inhibition of stromal-derived factor-1 (SDF1)/CXCR4 signaling, and impaired MSC migration [37]. MMP-2, MMP-14, and CXCR2 are expressed by MSCs to promote migration to injured tissues [38]. Valproic acid up-regulates the expression of CXCR4 and MMP-2, and promotes MSC migration [39]. Moreover, silencing MMP-2 inhibits the migratory activity of MSCs through bone marrow endothelium [40]. Likewise, silencing MMP-2 and MMP-14 impairs BM-MSC migration [41]. The up-regulation of MMP-2 and MMP-14 by various inflammatory cytokines is crucial for the recruitment of MSCs, and their migratory activities to injured tissues [41]. Our data showed that silencing MMP-2 and MMP-14 decreased the migratory activities of AMSCs during EC differentiation compared with AMSCs in EGM only. These findings indicate the crucial effect of MMP-2 and MMP-14 in AMSC migration, and provide additional key role of MMP-2 and MMP-14 in the re-endothelialization of denuded arteries following intervention.

Ang II induces the expression and activity of MMP-2 via the JNK pathway in macrovascular ECs, indicating the importance of ATRs in the activation of MMP-2 [42]. It has also been reported that Ang II induces the expression of MMP-2 and MMP-14 [43], through the MAPK pathway in epithelial cells [44]. Because ATR2 signals can increase the expression of MMP-2 and MMP-14, we examined the effect of silencing ATR2 on the migration of AMSCs during endothelial differentiation. Our data showed no significant difference in the migration of ATR2-silenced AMSCs compared with AMSCs with EGM only. These results indicate that ATR2 has no effect on the migration of AMSCs during endothelial differentiation, and therefore may have no effect on MMP-2 and MMP-14 activity or expression.

Ang II in combination with VEGF was found to induce BM-MSC differentiation to ECs, and blockage of ATR2 attenuated this process [27]. Stimulation of ATR2 was also found to activate neural differentiation, and monocyte differentiation to dendritic cells [28, 29]. However, in this study the data revealed that ATR2 silencing has no effect on the expression of EC markers after 10 days of stimulation for EC differentiation. Although the role of Ang II in the differentiation of MSCs has been reported in a few studies, the effect of ATR2 on the differentiation of MSCs to EC is not fully understood. Treatment with Ang II and VEGF was reported to enhance the EC differentiation of BM-MSCs through ATR2, whereas Ang II alone failed to induce differentiation [27]. In another study, incubation of MSCs with Ang II was found to increase the mRNA and protein expression of VEGF, and inhibition of ATR1, but not ATR2, impaired this effect [45]. Bone marrow-derived endothelial progenitor cells isolated from an Ang II-infusion rat model showed significant decrease in the differentiation compared with cells from untreated rats, suggesting a negative effect of Ang II on the differentiation [46]. The underlying mechanism of Ang II effect in the re-endothelialization process is not completely known. It is not clear whether Ang II promotes the EC differentiation by the induction of VEGF expression through ATR2. Moreover, MSCs derived from various sources respond differentially toward a specific culture treatment [47]. This may explain the absence of ATR2 effect on the differentiation of AMSCs into ECs in this study in comparison with its role in EC differentiation of BM-MSC [27]. Further in-vitro studies are needed to address the effect of Ang II on the differentiation of MSCs to ECs, and to determine whether ATR1 and ATR2 are involved in the induction of this process.

To date, little is known about the mechanisms underlying MSC differentiation to ECs. In a recent report, our data indicated that VEGFR2 is required for the EC differentiation of AMSCs. The increased expression of EC markers after silencing MMP-2 and MMP-14 was due to the cleavage activity of these proteases on the extracellular domains of VEGFR2 [8]. Inhibition of VEGFR2 kinase during EC differentiation resulted in significant decrease in the expression of EC markers, indicating that EC differentiation is regulated through VEGFR2 activity [8]. VEGFR2 is involved in most of the cellular activities of vascular ECs [18]. MAPK signal transduction pathways are initiated by either integrins or growth factors [48], and are known to have a key role in various cellular activities including proliferation and differentiation [49]. VEGFR2 signals are crucial for EC differentiation and survival [50]. Because VEGFR2, as a growth factor, regulates AMSC differentiation into ECs, MAPK may play a key role in mediating this differentiation.

To determine the downstream signaling of VEGFR2 during EC differentiation, we examined MAPK/ERK, JNK, and p38 signaling pathways. JNK phosphorylation significantly decreased after 10 days of stimulation for EC differentiation compared with nonstimulated cells (EBM), while p-p38 was not clearly affected under the same conditions. The phosphorylation of ERK, however, was significantly increased after 10 days of EC differentiation in EGM + MMP-2 siRNA and EGM + MMP-14 siRNA. This indicates that the change in ERK phosphorylation is in parallel with the increased activities of VEGFR2, suggesting that ERK receives signals from VEGFR2 and induces the transcription of EC markers during AMSC differentiation to ECs (Fig. 7). When ERK is activated, it can directly phosphorylate some transcription factors and induce differentiation [51]. Moreover, the results showed that the expression of EC markers (PECAM1 and VE-cadherin) significantly declined with inhibition of ERK during EC differentiation compared with stimulated AMSCs without ERK inhibitor. To further support our data demonstrating an association between VEGFR2 activity and ERK phosphorylation, a dose-dependent inhibition of ERK by VEGFR2 kinase inhibitor was performed. The results showed significant decrease in ERK phosphorylation associated with the increase in the concentration of VEGFR2 kinase inhibitor. These findings indicated that VEGF/VEGFR2 binding transmits signals to promote the differentiation of AMSCs into ECs through the activation of ERK pathway. During the stimulation of AMSCs for EC differentiation, the media were replaced every 3 days, which allows continuous stimulation for VEGF/VEGFR2-induced differentiation of AMSCs to ECs. Altogether, the results suggest that the EC differentiation of AMSCs is initiated via transmembrane signaling of VEGFR2 that turns on the downstream signaling of ERK/MAPK cascades and induces the expression of EC markers PECAM-1 and VE-cadherin.

Cellular stress and cytokines are the major stimuli that activate the JNK pathway and mediate apoptosis, proliferation, or cell survival [52]. The crosstalk inhibition between MAPK pathways is promoted by protein phosphatases. These phosphatases control cell responsiveness to a stimulus that promotes physiological functions, and prevent prolonged stimulation from causing pathological effects [52]. Inhibition of p38 has been reported to increase ERK phosphorylation, whereas ERK inhibition induces p38 phosphorylation and promotes osteogenic differentiation of BM-MSCs, indicating a crosstalk between ERK and p38 signaling pathways [53]. Another study implicated the essential role of JNK and p38 activation, but not ERK, in erythroid differentiation of SKT6 cells (Epo-responsive mouse erythroleukemia) [54]. Mixed lineage kinase-3 (MLK-3) is a serine/threonine kinase that activates the JNK signaling pathway [55]. MLK-3 restricts ERK phosphorylation independent of rapidly accelerated fibrosarcoma (Raf) activation [56]. This effect can be reversed by inhibition of JNK, suggesting a negative crosstalk between JNK and ERK signaling pathways [55]. Moreover, MAPK phosphatase-7 (MKP-7), a JNK-specific phosphatase, anchors p-ERK and prevents ERK-mediated transcription [57]. Additionally, p-ERK can phosphorylate and stabilize MKP-7, indicating that ERK activation blocks the JNK pathway [58, 59]. Our study demonstrated the inhibitory effect of ERK activation on JNK pathway (Fig. 7). The phosphorylation of ERK was significantly increased in EGM + MMP-2 siRNA and EGM + MMP-14 siRNA groups, which was also associated with significant decreases in p-JNK.

The current study provides clear evidence for the key role of the ERK signaling pathway in promoting the differentiation of AMSCs to ECs through VEGFR2. Such molecular insights provide new mechanistic strategies on how to enhance the rate of differentiating AMSCs to ECs. In addition, VEGFR2-specific agonists may provide a potential therapeutic strategy for the re-endothelialization of injured blood vessels. However, the ERK signaling pathway play a major role in various cellular events, and targeting this pathway with synthetic or natural mediators may interfere with other cellular processes including apoptosis. Because activation of the JNK pathway was implicated in osteogenesis and neuronal differentiation, but not EC differentiation, a synthetic inhibitor for JNK may also improve the commitment of the differentiating AMSCs specifically toward the EC lineage and prevent unwanted differentiation to other lineages. The better understanding of key signaling pathways that regulate AMSC differentiation to ECs would further enhance the potential use of AMSCs in regenerative medicine to treat cardiovascular diseases.

## Conclusion

In the present study, we demonstrate that VEGFR2-induced up-regulation of EC markers is mediated by the activation of ERK signaling pathway, whereas p38 and JNK had no effect on this process. The activation of ERK pathway resulted in a negative crosstalk with JNK pathway, and inhibited JNK phosphorylation. Hence, further in-vivo studies would support the findings. ATR2 silencing showed no effect on AMSC differentiation to ECs. These findings provide new insights into the role of VEGFR2 and the ERK signaling pathway in AMSC differentiation to ECs for the potential clinical uses of AMSCs. A large number of clinical trials with long-term follow up would enhance our understanding of the therapeutic efficiency of AMSCs in regenerative medicine.

## Abbreviations

AMSC: Adipose-derived mesenchymal stem cell
Ang II: Angiotensin II
ATR2: Angiotensin receptor R2
BSA: Bovine serum albumin
EBM: Endothelial cell basal medium
EC: Endothelial cell
EGM: Endothelial cell growth medium
ERK: Extracellular signal-regulated kinase
FBS: Fetal bovine serum
JNK: c-Jun NH2-terminal kinase
MKP-7: MAP kinase phosphatase-7
MLK: Mixed lineage kinase-3
MMP: Matrix metalloproteinase
p38: Stress activated protein kinase-2
PBS: Phosphate-buffered saline
VEGF: Vascular endothelial growth factor
VEGFR2: Vascular endothelial growth factor receptor-2
VSMC: Vascular smooth muscle cell

## References

[1] Scannapieco FA, Bush RB, Paju S (2003). Associations between periodontal disease and risk for atherosclerosis, cardiovascular disease, and stroke. A systematic review. Ann Periodontol.

[2] Deanfield JE, Halcox JP, Rabelink TJ (2007). Endothelial function and dysfunction: testing and clinical relevance. Circulation.

[3] Denes L, Entz L, Jancsik V (2012). Restenosis and therapy. Int J Vasc Med.

[4] In ’t Anker PS, Noort WA, Scherjon SA, Kleijburg-Van der Keur C, Kruisselbrink AB, Van Bezooijen RL (2003). Mesenchymal stem cells in human second-trimester bone marrow, liver, lung, and spleen exhibit a similar immunophenotype but a heterogeneous multilineage differentiation potential. Haematologica.

[5] Ringe J, Leinhase I, Stich S, Loch A, Neumann K, Haisch A, Häupl T, Manz R, Kaps C, Sittinger M (2008). Human mastoid periosteum-derived stem cells: promising candidates for skeletal tissue engineering. J Tissue Eng Regen Med.

[6] Pierdomenico L, Bonsi L, Calvitti M, Rondelli D, Arpinati M, Chirumbolo G (2005). Multipotent mesenchymal stem cells with immunosuppressive activity can be easily isolated from dental pulp. Transplantation.

[7] Ning H, Liu G, Lin G, Yang R, Lue TF, Lin C-S (2009). Fibroblast growth factor 2 promotes endothelial differentiation of adipose tissue-derived stem cells. J Sex Med.

[8] Almalki SG, Valle YL, Agrawal DK (2017). MMP-2 and MMP-14 silencing inhibits VEGFR2 cleavage and induces the differentiation of porcine adipose-derived mesenchymal stem cells to endothelial cells. Stem Cells Transl Med.

[9] Fearnley GW, Smith GA, Abdul-Zani I, Yuldasheva N, Mughal NA, Homer-Vanniasinkam S (2016). VEGF-A isoforms program differential VEGFR2 signal transduction, trafficking and proteolysis. Biol Open.

[10] Gomes E, Rockwell P (2008). p38 MAPK as a negative regulator of VEGF/VEGFR2 signaling pathway in serum deprived human SK-N-SH neuroblastoma cells. Neurosci Lett.

[11] Di Cristo G, Berardi N, Cancedda L, Pizzorusso T, Putignano E, Ratto GM (2001). Requirement of ERK activation for visual cortical plasticity. Science.

[12] McCubrey JA, Steelman LS, Hoyle PE, Blalock WL, Weinstein-Oppenheimer C, Franklin RA (1998). Differential abilities of activated Raf oncoproteins to abrogate cytokine dependency, prevent apoptosis and induce autocrine growth factor synthesis in human hematopoietic cells. Leukemia.

[13] Kasper G, Dankert N, Tuischer J, Hoeft M, Gaber T, Glaeser JD (2007). Mesenchymal stem cells regulate angiogenesis according to their mechanical environment. Stem Cells.

[14] Traverse S, Gomez N, Paterson H, Marshall C, Cohen P (1992). Sustained activation of the mitogen-activated protein (MAP) kinase cascade may be required for differentiation of PC12 cells—comparison of the effects of nerve growth-factor and epidermal growth-factor. Biochem J.

[15] Hsu C-L, Kikuchi K, Kondo M (2007). Activation of mitogen-activated protein kinase kinase (MEK)/extracellular signal regulated kinase (ERK) signaling pathway is involved in myeloid lineage commitment. Blood.

[16] Lu J, Liu K, Zhao J, Zhao J, Ma J, Yang H (2011). VEGF-A not Ang2 mediates endothelial-like differentiation of immature DCs by ERK1/2 signaling in the microenvironment of human colon adenocarcinoma. Int J Oncol.

[17] Lu J, Zhao J, Liu K, Zhao J, Yang H, Huang Y (2010). MAPK/ERK1/2 signaling mediates endothelial-like differentiation of immature DCs in the microenvironment of esophageal squamous cell carcinoma. Cell Mol Life Sci.

[18] Olsson A-K, Dimberg A, Kreuger J, Claesson-Welsh L (2006). VEGF receptor signalling—in control of vascular function. Nat Rev Mol Cell Biol.

[19] Xu J, Liu X, Jiang Y, Chu L, Hao H, Liu Z (2008). MAPK/ERK signaling mediates VEGF-induced bone marrow stem cell differentiation into endothelial cell. J Cell Mol Med.

[20] Bento LW, Zhang Z, Imai A, Nör F, Dong Z, Shi S (2013). Endothelial differentiation of SHED requires MEK1/ERK signaling. J Dent Res.

[21] Hu Y, Chan E, Wang SX, Li B (2003). Activation of p38 mitogen-activated protein kinase is required for osteoblast differentiation. Endocrinology.

[22] Zhao Y, Song T, Wang W, Wang J, He J, Wu N, et al. P38 and ERK1/2 MAPKs act in opposition to regulate BMP9-induced osteogenic differentiation of mesenchymal progenitor cells. PLoS One. 2012;7(8):e43383. doi: 10.1371/journal.pone.0043383. 10.1371/journal.pone.0043383 PMC3422272 22912865

[23] Tiwari VK, Stadler MB, Wirbelauer C, Paro R, Schübeler D, Beisel C (2012). A chromatin-modifying function of JNK during stem cell differentiation. Nat Genet.

[24] Wei ZZ, Yu SP, Lee JH, Chen D, Taylor TM, Deveau TC (2014). Regulatory role of the JNK-STAT1/3 signaling in neuronal differentiation of cultured mouse embryonic stem cells. Cell Mol Neurobiol.

[25] Zhang X, Zhou C, Zha X, Xu Z, Li L, Liu Y (2015). Apigenin promotes osteogenic differentiation of human mesenchymal stem cells through JNK and p38 MAPK pathways. Mol Cell Biochem.

[26] Ahmadian E, Jafari S, Yari KA (2015). Role of angiotensin II in stem cell therapy of cardiac disease. J Renin Angiotensin Aldosterone Syst.

[27] Ikhapoh I, Pelham C, Agrawal D (2015). Synergistic effect of angiotensin II on vascular endothelial growth factor-A-mediated differentiation of bone marrow-derived mesenchymal stem cells into endothelial cells. Stem Cell Res Ther.

[28] Guimond M-O, Gallo-Payet N (2012). The angiotensin II type 2 receptor in brain functions: an update. Int J Hypertens.

[29] Guimond MO, Gallo-Payet N (2012). How does angiotensin AT2 receptor activation help neuronal differentiation and improve neuronal pathological situations?. Front Endocrinol (Lausanne).

[30] Kumar G, Hara H, Long C, Shaikh H, Ayares D, Cooper DKC (2012). Adipose-derived mesenchymal stromal cells from genetically modified pigs: immunogenicity and immune modulatory properties. Cytotherapy.

[31] Hass R, Kasper C, Böhm S, Jacobs R (2011). Different populations and sources of human mesenchymal stem cells (MSC): a comparison of adult and neonatal tissue-derived MSC. Cell Commun Signal.

[32] Baer PC (2014). Adipose-derived mesenchymal stromal/stem cells: an update on their phenotype in vivo and in vitro. World J Stem Cells.

[33] Vieira NM, Brandalise V, Zucconi E, Secco M, Strauss BE, Zatz M (2010). Isolation, characterization, and differentiation potential of canine adipose-derived stem cells. Cell Transplant.

[34] Almalki SG, Agrawal DK. Effects of matrix metalloproteinases on the fate of mesenchymal stem cells. Stem Cell Res Ther. 2016;7(1):129. doi: 10.1186/s13287-016-0393-1. 10.1186/s13287-016-0393-1 PMC5016871 27612636

[35] Manduca P, Castagnino A, Lombardini D, Marchisio S, Soldano S, Ulivi V (2009). Role of MT1-MMP in the osteogenic differentiation. Bone.

[36] Rao VH, Rai V, Stoupa S, Subramanian S, Agrawal DK. Tumor necrosis factor-alpha regulates triggering receptor expressed on myeloid cells-1-dependent matrix metalloproteinases in the carotid plaques of symptomatic patients with carotid stenosis. Atherosclerosis. 2016;248:160-9. 10.1016/j.atherosclerosis.2016.03.021 PMC4836960 27017522

[37] Bhoopathi P, Chetty C, Gogineni VR, Gujrati M, Dinh DH, Rao JS (2011). MMP-2 mediates mesenchymal stem cell tropism towards medulloblastoma tumors. Gene Ther.

[38] Son BR, Marquez-Curtis LA, Kucia M, Wysoczynski M, Turner AR, Ratajczak J (2006). Migration of bone marrow and cord blood mesenchymal stem cells in vitro is regulated by SDF-1-CXCR4 and HGF-c-met axes and involves matrix metalloproteinases. Stem Cells.

[39] Marquez-Curtis LA, Qiu Y, Xu A, Janowska-Wieczorek A. Migration, proliferation, and differentiation of cord blood mesenchymal stromal cells treated with histone deacetylase inhibitor valproic acid. Stem Cells Int. 2014;2014:610495. doi: 10.1155/2014/610495. 10.1155/2014/610495 PMC3976771 24757448

[40] De Becker A, Van Hummelen P, Bakkus M, Vande BI, De Wever J, De Waele M (2007). Migration of culture-expanded human mesenchymal stem cells through bone marrow endothelium is regulated by matrix metalloproteinase-2 and tissue inhibitor of metalloproteinase-3. Haematologica.

[41] Ries C, Egea V, Karow M, Kolb H, Jochum M, Neth P (2007). MMP-2, MT1-MMP, and TIMP-2 are essential for the invasive capacity of human mesenchymal stem cells: differential regulation by inflammatory cytokines. Blood.

[42] Jiménez E, de la Blanca EP, Urso L, González I, Salas J, Montiel M (2009). Angiotensin II induces MMP 2 activity via FAK/JNK pathway in human endothelial cells. Biochem Biophys Res Commun.

[43] Striker GE, Praddaude F, Alcazar O, Cousins S, Marin-Castaño M (2008). Regulation of angiotensin II receptors and extracellular matrix turnover in human retinal pigment epithelium: role of angiotensin II. Am J Physiol Cell Physiol.

[44] Pons M, Cousins SW, Alcazar O, Striker GE, Marin-Castaño ME (2011). Angiotensin II-induced MMP-2 activity and MMP-14 and basigin protein expression are mediated via the angiotensin II receptor type 1-mitogen-activated protein kinase 1 pathway in retinal pigment epithelium: implications for age-related macular degeneration. Am J Pathol.

[45] Shi R-Z, Wang J-C, Huang S-H, Wang X-J, Li Q-P (2009). Angiotensin II induces vascular endothelial growth factor synthesis in mesenchymal stem cells. Exp Cell Res.

[46] Kobayashi K, Imanishi T, Akasaka T (2006). Endothelial progenitor cell differentiation and senescence in an angiotensin II-infusion rat model. Hypertens Res.

[47] Yun CH (2014). The impact of mesenchymal stem cell source on proliferation, differentiation, immunomodulation and therapeutic efficacy. J Stem Cell Res Ther.

[48] Lai CF, Chaudhary L, Fausto A, Halstead LR, Ory DS, Avioli LV (2001). Erk is essential for growth, differentiation, integrin expression, and cell function in human osteoblastic cells. J Biol Chem.

[49] Atay O, Skotheim JM (2017). Spatial and temporal signal processing and decision making by MAPK pathways. J Cell Biol.

[50] Sase H, Watabe T, Kawasaki K, Miyazono K, Miyazawa K (2009). VEGFR2-PLCgamma1 axis is essential for endothelial specification of VEGFR2+ vascular progenitor cells. J Cell Sci.

[51] Li Z, Theus MH, Wei L (2006). Role of ERK 1/2 signaling in neuronal differentiation of cultured embryonic stem cells. Dev Growth Differ.

[52] Junttila MR, Li S-P, Westermarck J (2008). Phosphatase-mediated crosstalk between MAPK signaling pathways in the regulation of cell survival. FASEB J.

[53] Thi Kim Phuong D, Kyung Soon P, Hyung Keun K, Dae Sung P, Ji Hyun Kim TRY. Inhibition of JNK and ERK pathways by SP600125- and U0126-enhanced osteogenic differentiation of bone marrow stromal cells. Tissue Eng Regen Med. 2012;9:283–94.

[54] Nagata Y, Takahashi N, Davis RJ, Todokoro K (1998). Activation of p38 MAP kinase and JNK but not ERK is required for erythropoietin-induced erythroid differentiation. Blood.

[55] Shen YH, Godlewski J, Zhu J, Sathyanarayana P, Leaner V, Birrer MJ (2003). Cross-talk between JNK/SAPK and ERK/MAPK pathways: sustained activation of JNK blocks ERK activation by mitogenic factors. J Biol Chem.

[56] Brancho D, Ventura J-J, Jaeschke A, Doran B, Flavell RA, Davis RJ (2005). Role of MLK3 in the regulation of mitogen-activated protein kinase signaling cascades. Mol Cell Biol.

[57] Masuda K, Katagiri C, Nomura M, Sato M, Kakumoto K, Akagi T (2010). MKP-7, a JNK phosphatase, blocks ERK-dependent gene activation by anchoring phosphorylated ERK in the cytoplasm. Biochem Biophys Res Commun.

[58] Masuda K, Shima H, Katagiri C, Kikuchi K (2003). Activation of ERK induces phosphorylation of MAPK phosphatase-7, a JNK specific phosphatase, at Ser-446. J Biol Chem.

[59] Katagiri C, Masuda K, Urano T, Yamashita K, Araki Y, Kikuchi K (2005). Phosphorylation of Ser-446 determines stability of MKP-7. J Biol Chem.

